# Publicly available *ex vivo* transcriptomics datasets to explore CNS physiology and neurodegeneration: state of the art and perspectives

**DOI:** 10.3389/fnins.2023.1211079

**Published:** 2023-08-23

**Authors:** Sandra Abdullatef, Cinthia Farina

**Affiliations:** ^1^Division of Neuroscience, Institute of Experimental Neurology (INSpe), IRCCS San Raffaele Scientific Institute, Milan, Italy; ^2^Faculty of Medicine, Università Vita-Salute San Raffaele, Milan, Italy

**Keywords:** age, glial cells, neurodegeneration, neurons, physiology, RNA-Seq, sex, transcriptomics

## Abstract

The central nervous system (CNS) is characterized by an intricate composition of diverse cell types, including neurons and glia cells (astrocytes, oligodendrocytes, and microglia), whose functions may differ along time, between sexes and upon pathology. The advancements in high-throughput transcriptomics are providing fundamental insights on cell phenotypes, so that molecular codes and instructions are ever more described for CNS physiology and neurodegeneration. To facilitate the search of relevant information, this review provides an overview of key CNS transcriptomics studies ranging from CNS development to ageing and from physiology to pathology as defined for five neurodegenerative disorders and their relative animal models, with a focus on molecular descriptions whose raw data were publicly available. Accurate phenotypic descriptions of cellular states correlate with functional changes and this knowledge may support research devoted to the development of therapeutic strategies supporting CNS repair and function.

## Background

The central nervous system (CNS) is characterized by large heterogeneity of cellular components organized in complex and plastic circuits evolved to sustain information processing and regulate vital body functions, including breathing, language, cognition, and memory (Martinez and Sprecher, 2020). It is known that CNS composition and function differentiates along time, between sexes and upon pathology (Larson, 2018). The advancements in high-throughput transcriptomics methods are providing fundamental insights on glial and neuronal phenotypes, so that molecular codes and instructions are ever more described for CNS physiology and its pathological states in neurodegenerative disorders, where neuronal damage is not fully autonomous but represents the final step of a series of events involving glia–glia and glia–neuron interactions. Thus, functional changes may be highlighted by accurate phenotypic descriptions of cellular states and this knowledge may support research devoted to the development of therapeutic strategies supporting CNS repair and function.

**Figure 1:**
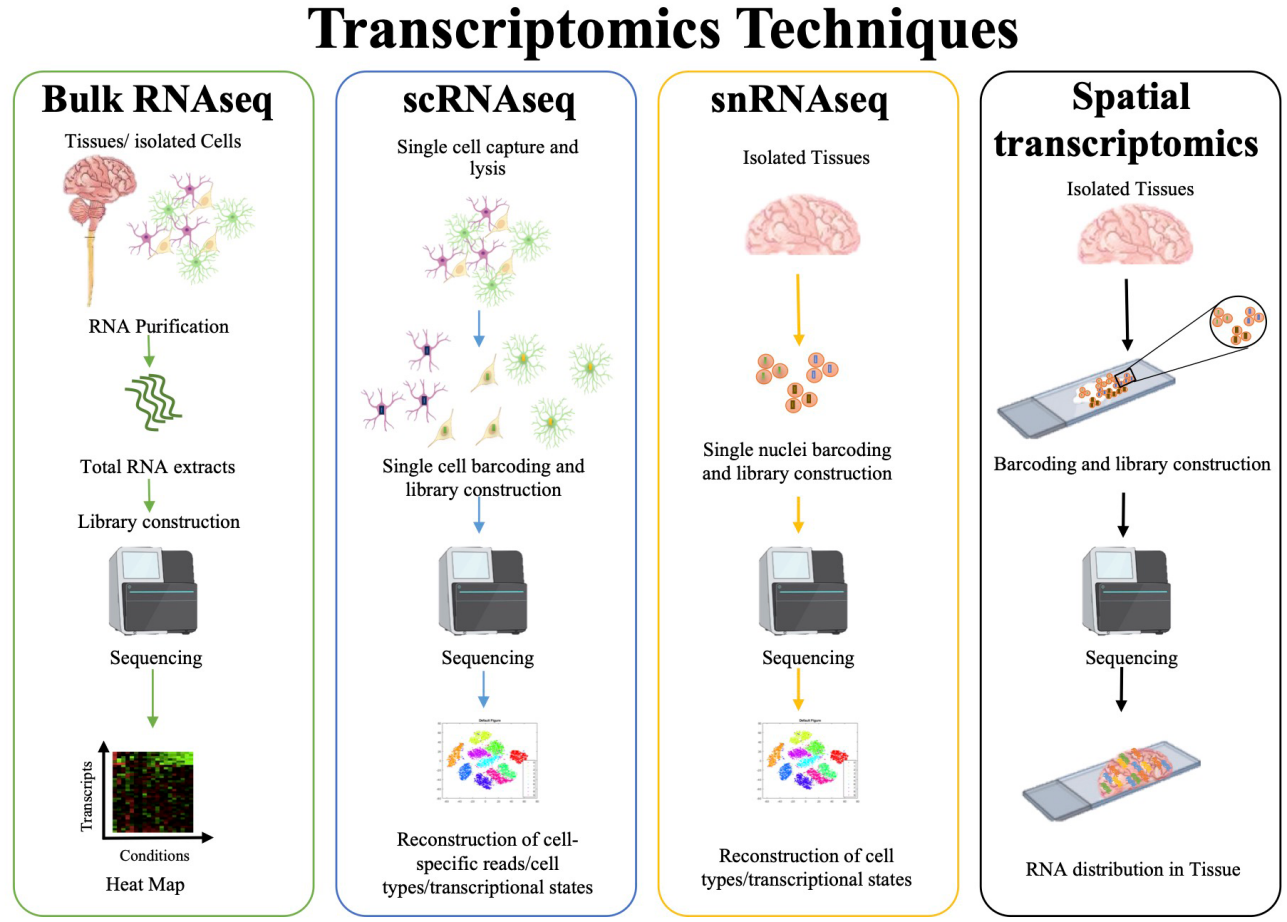
RNA sequencing workflows.

Relevant information on CNS state has been collected via a variety of molecular techniques, from bulk to spatial transcriptomics. Early pioneering studies using bulk transcriptomics generated for example initial public databases for CNS cell types (Cahoy et al., 2008; Doyle et al., 2008), defined miRNAs promoting myelin development (Dugas et al., 2010), and identified targets for tissue repair (Huang et al., 2011). Bulk RNA profiling provides widespread and reliable quantitative insights into the gene expression of a whole tissue or a large number of samples at low cost (Saliba et al., 2014; Li and Wang, 2021). However, bulk transcriptomics measurements average global gene expression and fail to identify the cellular source of signals, a limit that has been breached by single cell or single nucleus RNA sequencing (sc or snRNAseq; Figure 1). These techniques may offer unprecedented insights into development, heterogeneity and dynamics of distinct cell types within a tissue, however they display high costs and limits in sample number under analysis and sequencing depth (Li and Wang, 2021). Further, snRNA-seq, which does have the advantage to get information at the single cell level from frozen tissues and is free from artifacts resulting from the dissociation protocols (Lacar et al., 2016), generates information on immature transcripts and misses mitochondrial and cytoplasmic transcripts. A well-known technique useful to locate transcripts within the tissues is *in situ* hybridization (ISH), where target mRNAs are imaged for example via fluorescently labeled, gene-specific probes (Williams et al., 2022). The last frontier in spatial transcriptomics applies untargeted RNA-sequencing to barcoded positional information, thus providing spatial representation of several transcripts within the tissue at the same time. Its downsides are non-single cell resolution, relatively low sensitivity, high costs and labor intensive processes (Williams et al., 2022).

This review provides an overview of representative CNS transcriptomics studies from development to ageing, from physiology to pathology as described for five neurodegenerative disorders (multiple sclerosis MS, Alzheimer’s disease AD, amyotrophic lateral sclerosis ALS, Parkinson’s disease PD and Huntington disease HD) and their mouse models (see Box 1 for description of diseases and models). Literature search was conducted in PubMed using transcriptome and CNS or specific cell type names as keywords and including studies published between January 2012 till May 2022. We selected representative original articles describing *ex vivo* transcriptomics data for *Mus musculus* and *Homo sapiens* whose raw datasets were publicly available. Datasets were ordered according to whether they referred to whole tissue or cell type-specific analyses under physiological or pathological states. The results of our search are summarized in Table 1, which reports dataset identification codes divided according to input tissue and technique (whole tissues transcriptomes as analyzed by bulk RNA-seq, cell type specific transcriptomes as analyzed by bulk RNA-seq of purified CNS cell types or sc/snRNA-seq experiments of CNS tissues or purified cells), species (*Homo sapiens* or *Mus musculus*), and physiological or pathological states (Healthy CNS, MS, AD, PD, ALS and HD).

**Table 1:** Summary of CNS transcriptomic studies.

	Whole tissue		Oligodendrocytes		Astrocytes		Neurons		Microglia	
	*Homo sapiens*	*Mus musculus*	*Homo sapiens*	*Mus musculus*	*Homo sapiens*	*Mus musculus*	*Homo sapiens*	*Mus musculus*	*Homo sapiens*	*Mus musculus*
Healthy tissue	GSE63060 GSE68559 GSE97930 GSE60863 GSE67333 SRX034874 GSE26927 GSE140231 GSE11882	GSE79238 PRJNA510761 phs000833.v3.p1 GSE155081 GSE158380 GSE75330 GSE153164	GSE9566 GSE104276 GSE30272 GSE25219 GSE97942 GSE130105 GSE120046 GSE97930	GSE180604 GSE95093 GSE95194 GSE63060 GSE83931 GSE190399 GSE188646	GSE73721 GSE9566 GSE104276 GSE30272 GSE25219 GSE73721GSE130119	GSE63060 GSE83931 GSE192490 GSE114000 GSE72826 GSE152222 GSE147119	GSE9566 GSE104276 GSE30272 GSE25219 GSE97942 GSE130105 syn2580853 GSE120046 GSE97930	GSE143161 GSE142654 GSE184484 GSE40438 GSE63060 GSE83931 GSE190399	GSE9566 GSE104276 GSE30272 GSE25219 GSE97942 GSE130105 GSE120046 GSE135618 syn11468526	GSE63060 GSE83931 GSE137028 GSE190399 GSE188646 GSE9566 GSE165555 GSE161340 GSE135618
MS	GSE26927 GSE138614 GSE123496 GSE126802 PRJNA544731 phs000833.v3.p1 GSE5281 GSE48350	GSE172083 GSE131854 GSE154228 GSE166179	GSE179590 GSE129762 GSE118257	GSE113973 GSE118451 GSE178085 GSE154175	GSE179590 GSE129762 PRJNA544731 GSE100330	GSE149135 GSE155711 GSE149105 GSE100330 GSE136358 GSE100294	GSE179590 PRJNA544731 GSE118257	GSE161654 GSE178085	GSE141862 GSE179590 GSE111972 GSE124335 GSE129762	GSE149135 GSE155711 GSE149105 GSE185044
AD	GSE39420 GSE106241 GSE63061 GSE67333 GSE67333 GSE53697 GSE110720 GSE26927	GSE80437 GSE157161 GSE157766 GSE104775	GSE140511 syn18485175 GSE138852	GSE140511 syn21125841 GSE182762 GSE98971	GSE140511 phs000745.v1.p1 GSE138852	GSE140511 GSE182762 GSE98971	GSE140511 GSE138852 GSE110732 GSE4757	GSE140511 GSE182762 GSE183323 GSE98971	GSE140511 GSE138852 syn10934660 syn11209141 syn3159438 syn7392158 syn3157743 syn4645334 syn10901600 syn5759470	GSE104775 GSE127893 GSE123467 GSE103334
PD	GSE7621 phs000833.v3.p1 GSE49036 GSE8397 GSE26927 GSE184484	GSE31458 GSE19534 GSE4788 GSE205907	GSE202210 GSE157783	CNP0000892	GSE202210 GSE157783	GSE191131 CNP0000892	GSE202210 GSE157783 GSE182622	CNP0000892	GSE202210 GSE157783 https://github.com/smukher2/GithubFrontiersNeurosciDec2018	GSE186559 GSE157533 GSE157534 GSE186483 CNP0000892 https://github.com/smukher2/GithubFrontiersNeurosciDec2018
ALS	GSE179819 GSE103225 GSE139900 phs000833.v3.p1 phs000747	GSE160402 GSE52118 GSE113924	GSE174332	GSE120374 GSE178693 GSE133047 GSE173524 GSE111031	GSE174332	GSE111031 GSE26927 GSE173524 GSE120374 GSE178693 https://als-st.nygenome.org	GSE40438 GSE76220 GSE40438 GSE173115 GSE132972 GSE174332	GSE173524 GSE142654 GSE184484 GSE120374 GSE178693 GSE38820 GSE166307 GSE111031	GSE174332 https://als-st.nygenome.org	GSE111031 GSE173524 GSE101689 GSE178693 GSE120374 https://als-st.nygenome.org GSE103607
HD	GSE26927 phs000833.v3.p1	GSE107613 GSE67829 GSE67761 GSE78274 GSE88920 PRJNA510761 GSE124846 GSE165658	GSE180928 GSE152058	GSE180928	GSE154128 GSE154141 GSE152058 GSE180928	GSE154131 GSE154141 PRJNA510761 GSE154128 GSE152058	GSE154128 GSE180928 GSE152058 https://vmenon.shinyapps.io/hd_sn_rnaseq/	GSE154128 GSE152058 GSE171099	GSE180928 GSE152058 https://github.com/smukher2/GithubFrontiersNeurosciDec2018	GSE154131 GSE152058 https://github.com/smukher2/GithubFrontiersNeurosciDec2018

Whole tissue column includes bulk RNA-seq studies of CNS tissues under health and disease. Cell type specific columns include bulk RNA-seq of purified CNS cell types or sc/snRNA-seq experiments of CNS tissues/cells.

## Transcriptomics of CNS tissues under physiology and disease

RNAseq of tissues from multiple brain regions and neocortical areas of developing and adult brain highlighted spatiotemporal heterogeneity and sex-related features in gene expression of the human brain (Kang et al., 2011; Table 1, GSE25219). Regional diversity may regard both long non coding RNAs and mRNAs (Webb et al., 2015; Table 1, GSE68559). Regional and sex differences were described for the mouse CNS as well (Bundy et al., 2017; Table 1, GSE83931). Bulk transcriptomics of human *post mortem* tissues relative to 269 subjects surveyed temporal dynamics in genome expression in neural tissue from fetal development to ageing and highlighted gene expression changes occurring during fetal life which were reversed in early postnatal life and then reacquired with ageing and neurodegeneration (Colantuoni et al., 2011; Table 1, GSE30272). An accurate overview of described transcriptome analyses of human brain ageing underlined that aging is associated with loss in synaptic function and acquisition of innate immune functions (Ham and Lee, 2020).

A recent review summarized CNS transcriptomics studies in multiple sclerosis (Elkjaer et al., 2022). For example, RNA-seq of a large number of white matter MS lesions provided molecular profiling for distinct lesion types and identified TGFBR2 as major common hub mostly upregulated in remyelinating lesions (Elkjaer et al., 2019; Table 1, GSE138614). Alzheimer’s disease and asymptomatic AD (a condition referred to individuals with intact cognition but neuropathology consistent with AD) were found to be characterized by common changes in frontal cortex transcriptome, involving, e.g., genes playing a role in astrocyte glutamate-glutamine cycle, and by differences encompassing transcripts related to stress response and removal of amyloidogenic proteins in AD (Patel et al., 2019; Table 1, GSE118553). PD progression, as assessed at the level of substantia nigra, found also transcriptomics descriptions, including deregulation of pathways linked to axonal degeneration and immunity in Braak stages 1 and 2 to alterations in dopaminergic signaling in Braak stages 5 and 6 (Dijkstra et al., 2015; Table 1, GSE49036). Further, it was characterized by dysregulation in transcripts involved in B cell and T cell signaling, suggesting the involvement of adaptive immunity in this disorder (Dijkstra et al., 2015; Table 1, GSE49036). Transcriptional changes after exercise were described in substantia nigra (SN) and striatum in the MPTP-induced PD model (Tong et al., 2022; Table 1, GSE205907). Brain RNA-seq highlighted dysregulation of ribosomal genes and led to the definition of stress granule formation in a mouse model for C9orf72 ALS (Table 1, GSE112931). Transcriptomics studies of human motor cortex from HD patients and controls revealed aberrant expression of genes involved in splicing, including PTBP1 (Lin et al., 2016; Table 1, GSE79666).

## Transcriptomics of oligodendrocytes under physiology and disease

Oligodendrocytes provide metabolic support to neurons and build myelin sheaths around axons, thus making conduction of action potential efficient (Philips and Rothstein, 2017). Neurons, oligodendrocytes, and astrocytes derive from a common multipotent self-renewable neural stem cell in a process that occurs with precise timing. While neurogenesis takes place early during embryonic development and is accomplished at about birth, gliogenesis follows neurogenesis and is finalized in postnatal life (Freeman, 2010), with synaptogenesis and neuronal function depending on glial maturation (Stevens, 2008). Accordingly, bulk and single cell RNA profiling of oligodendrocyte precursor cells (OPC) isolated from rodent embryos (E13.5) or postnatal mice (P7) evidenced transcriptional signatures emerging at P7 which were linked with differentiation and mostly convergent between spinal cord and brain cells (Marques et al., 2018; Table 1, GSE95194, GSE95093). A scRNA sequencing study of oligodendrocyte lineage cells from 10 regions of the mouse juvenile (P21-P30) and adult (P60) CNS was unable to identify region- or age-specific subpopulations of OPC, however distinct adult CNS regions were populated by diverse mature oligodendrocytes some of which could be found already in juvenile CNS, suggesting specific regional and temporal propensity to final myelination (Marques et al., 2016; Table 1, GSE75330).

Sexual dimorphism is reported for rodent oligodendrocytes and regards OL density in brain and spinal cord, myelin protein content and OL turnover (Cerghet et al., 2006). While sex-specific transcriptional profiles were detailed for cultured oligodendrocyte precursors (Yasuda et al., 2020), the description of the transcriptional phenotype of freshly isolated myelin-forming cells in two sexes remains an issue to be investigated along development and ageing.

CNS disorders may be characterized by alterations in OL number and phenotype. MS may present with different types of pathological lesions for which OL heterogeneity has been evidenced by snRNAseq (Jäkel et al., 2019). Human OL clusters did show some similarities to adult mouse counterparts and cluster abundance changed across MS lesions (Jäkel et al., 2019; Table 1, GSE118257). A single cell transcriptomics study of EAE spinal cord and cerebellum highlighted major expression of genes involved in antigen presentation (e.g., MHC-I and II, B2m, Psmb9, Tap1 and Tap2) in OL during neuroinflammation which was confirmed in human tissues (Falcão et al., 2018; Table 1, GSE113973). Indeed, *in vitro* studies demonstrated that MHC-II-expressing OL can present antigen and activate effector CD4 positive T cells, suggesting that OLs in MS may support immune responses also *in vivo* (Falcão et al., 2018). snRNAseq studies of human post-mortem prefrontal cortex of AD patients and control subjects revealed OL clusters correlating with disease (Mathys et al., 2019; Zhou et al., 2020; Table 1, syn18485175, GSE140511). Oligodendrocytes showed reduced expression of genes SEMA3B, STMN4, and MIR219A2 that regulate maturation of myelin-forming cells, axon guidance, and actin cytoskeleton rearrangements respectively, while upregulating expression of gene products sensitive to changes in pH and electrolyte levels (CA2), osmotic imbalances (SLC38A2), lipid accumulation (MID1IP1), and oxidative stress (SEPP1), probably as response to the accumulation of degradation products resulting from axonal degeneration (Zhou et al., 2020). In addition, snRNAseq brain data of aged 5XFAD and wild-type mice confirmed disease-associated alterations in OL state (Zhou et al., 2020; Table 1, syn21125841). Sex-specific transcriptional changes in CNS cells including OLs were described in AD, with male AD subjects showing global transcriptional activation in OL correlating with increased pathology (Mathys et al., 2019; Table 1, syn18485175). A snRNAseq comparative study of the human superior frontal gyrus across neurodegenerative diseases showed great differences in transcriptomic profiles of newly formed and mature OLs across MS, AD, and PD (Itoh and Voskuhl, 2017; Table 1, GSE26927, GSE8397, GSE48350). scRNAseq of cells isolated from the brainstem of symptomatic SOD1 mice and wildtype counterparts depicted transcriptional changes in ALS OLs for genes involved in neurogenesis, CNS development, and ensheathment of neurons (Liu et al., 2020), thus implying a role for oligodendrocytes also in this disorder (Table 1, GSE178693). Molecular pathology in the cortex and striatum from R6/2 mice and human HD tissues by snRNAseq highlighted deficits in OL maturation (Lim et al., 2022; Table 1, GSE180928, GSE180294).

Amplification, migration, and differentiation of OPC at injury site may be necessary for tissue repair (David-Bercholz et al., 2021). This mechanism can be properly assessed using the cuprizone model (see Box 1). The cuprizone-rich diet in fact activates CNS cells as astrocytes and leads to demyelination and oligodendrocyte loss (Colombo et al., 2021). Suspension of cuprizone diet allows for time-controlled analyses of remyelination processes after injury (Lassmann and Bradl, 2017). OL transcriptome during repair was assessed in the remyelination phase of the cuprizone model, and in EAE after treatment with estrogen receptor-β ligand, which induces remyelination. As a result both models displayed the upregulation of cholesterol synthesis, a pathway essential for myelination (David-Bercholz et al., 2021; Lim et al., 2022; Table 1, GSE118451). Importantly, a therapeutic regimen with estrogen receptor β-ligand during the remyelination phase of the cuprizone model further increased cholesterol-synthesis pathways and enhanced remyelination compared with vehicle treatment (Voskuhl et al., 2019). Similarly, estrogen receptor β-ligand treatment in the EAE model increased cholesterol-synthesis pathway gene expression in oligodendrocytes and induced remyelination (Voskuhl et al., 2019), thus indicating estrogen receptor β signaling as therapeutic target to increase cholesterol-synthesis pathways in OL and support remyelination.

## Transcriptomics of astrocytes under physiology and disease

Astrocytes offer vital homeostatic support to the CNS tissue as they secrete trophic factors, regulate ion and water balance in the extracellular milieau, modulate synapse formation, and are integral components of the blood–brain barrier (Colombo and Farina, 2016). Upon CNS injury, astrocytes become reactive, consequently proliferate, increase in size, and form a scar to limit tissue damage and support repair (Colombo and Farina, 2016; Escartin et al., 2021). SnRNAseq of astrocytes isolated from mouse striatum or cortex at P3, when they are still immature, or at adult age, when they have completed their differentiation, identified several maturation markers shared between the two brain regions, including, e.g., glutamate transporters (Lattke et al., 2021; Table 1, GSE152223). Regional specification was described for postnatal (P7) spinal cord astrocytes, with ventral glia cells expressing Semaphorin3a and thus providing positional clues important for proper motor neuron and sensory neuron circuit organization (Molofsky et al., 2014; Table 1, GSE55054). Regarding the adult stage, scRNA-seq of the mouse cortex and hippocampus also unraveled specialization of astrocytes between and within areas (Batiuk et al., 2020; Table 1, GSE114000). Ageing-associated transcriptional signatures were described for the mouse (Pan et al., 2020; Table 1, GSE137028), and some were shared among CNS areas, while others being region-specific (Boisvert et al., 2018; Table 1, GSE99791). Interestingly, aged astrocytes maintained expression of genes important for their homeostatic functions but acquired markers associated with reactive states during neuroinflammation (Boisvert et al., 2018; Clarke et al., 2018; Pan et al., 2020; Table 1, GSE137028, GSE99791, PRJNA417856).

Sex differences are described for astrocyte morphology, number, gene expression and function (Chowen and Garcia-Segura, 2021). Mouse postnatal cortical development displayed distinct timing and trajectory of transcriptional patterns between male and female astrocytes, suggesting that astroglia mature faster in male than female mice (Rurak et al., 2022; Table 1, GSE192490).

Neurodegenerative processes may alter astrocyte phenotype and, consequently, function. Cellular hypertrophy and GFAP expression are the most commonly used tools used to determine the reactive state of astrocytes in pathological specimens, but the advancement in transcriptomics allows to define astrocyte phenotypes at higher magnification. SnRNAseq of MS lesions revealed distinct expression patterns for cortical vs. subcortical reactive astrocytes, with white matter astrocytes strongly expressing GFAP and CD44, a molecule important for T cell differentiation and BBB permeability (Dzwonek and Wilczynski, 2015), and cortical astrocytes downregulating genes involved in glutamate and potassium homeostasis, which may have a detrimental impact on neuronal function and survival (Schirmer et al., 2019; Table 1, PRJNA544731). RNA-seq analysis across multiple regions of the CNS under physiology or EAE depicted transcriptional upregulation of genes involved in antigen presentation and downregulation of cholesterol synthesis genes in spinal cord and cerebellum astrocytes of EAE mice (Itoh et al., 2018; Table 1, GSE100330). *Wheeler* et al. described changes in distinct astrocyte clusters during EAE and validated the protective role of astrocytes expressing genes under transcriptional control of the transcription factor NRF2 and the pathogenic role of astrocytes under control of the transcription factor MAFG (Wheeler et al., 2020; Table 1, GSE130119). Transcriptomic analyses of astrocytes purified from optic nerves of EAE or control mice depicted major changes in levels of the inflammatory mediator complement component 3 or of the neuroprotective factor thrombospondin 1 in female or male astrocytes, respectively, (Tassoni et al., 2019; Table 1, GSE100294). ScRNAseq analysis of human AD highlighted astrocyte phenotypes distinct from those commonly found under neuroinflammation. In fact, AD brain was characterized by the contraction in a subpopulation of astrocytes evident in control tissue and enriched for genes involved in lipid and oxidative metabolism (Zhou et al., 2020; Table 1, GSE140511). SnRNAseq of prefrontal cortex from PD and age-matched control individuals demonstrated the upregulation of pathways related to detoxification of heavy metals in PD astrocytes compared to controls (Zhu et al., 2022; Table 1, GSE202210). Glial activation characterizes also ALS and its mouse SOD1 G93A model (Lei et al., 2019). Phenotypic alterations were found in SOD1 G93A spinal cord where astrocytes presented transcriptional changes in genes involved in complement activation and lipid metabolism (MacLean et al., 2022; Table 1, GSE173524), and in SOD1 G93A cortex, where astrocytes displayed dysregulation in genes associated with ion homeostasis and Wnt signaling (Miller et al., 2018; Table 1, GSE111031). Huntington Disease is characterized by several transcriptional changes in astrocytes, with shared alterations between human and mouse HD in genes involved in calcium dependent processes and glutamate receptor signaling (Merienne et al., 2019; Table 1, PRJNA510761).

## Transcriptomics of neurons under physiology and disease

Diversity in neuronal lineages from progenitor cells to mature excitatory or inhibitory neurons is evidenced in some transcriptomic studies reviewed in Vinsland and Linnarsson (2022). For example, scRNAseq of different regions of mouse brain during development (E7-E18) revealed that neuronal diversity was generated in post-mitotic neuroblasts and maturing neurons (La Manno et al., 2021; Table 1, PRJNA637987). ScRNAseq experiments complemented with spatial transcriptomics portrayed mouse corticogenesis from E10.5 to P4, so that neuronal differentiation and specification for the distinct cortical layers finds an accurate description at molecular level (Di Bella et al., 2021; Table 1, GSE153164). Single cell transcriptome profiling of the four cortical lobes and pons during human embryonic and fetal development highlighted spatio-temporal patterns from gestational week 9 to 28 (Fan et al., 2020; Table 1, GSE120046). Neurogenesis is a process occurring all over life but becomes restricted to subventricular zone and hippocampus in post-natal brain. scRNAseq of distinct areas of adult mouse subventricular zone demonstrated heterogeneity of neural precursor cells which may bear molecular positioning information for dorsal and ventral territories (Cebrian-Silla et al., 2021; Table 1, GSE165555). Further, neurogenesis in mouse hippocampus generates neurons which were classified into distinct maturation subgroups according to their expression profiles by scRNAseq (Gao et al., 2017; Table 1, GSE75901). Regarding neuronal specification, bulk RNA of distinct cell types purified from mouse forebrain at different postnatal stages (P1 and P30) combined with *in situ* hybridization demonstrated that several widely used neuronal markers, such as Map2 and Tau, are not exclusively expressed by neurons and that many neuron-specific genes (e.g., neurofilament chains L, M, and H) are expressed only by subsets of neurons (Cahoy et al., 2008; Table 1, GSE9566). Multilayered RNA-seq analysis depicted neuronal molecular specialization in the mouse hippocampus (Ha et al., 2021). Ageing is accompanied by transcriptional changes in hypothalamic mouse neurons, with peculiar alterations in X chromosome inactivation center genes in females. (Hajdarovic et al., 2022; Table 1, GSE188646).

Scientific literature also offers evidence of molecular correlates for neurodegenerative conditions. For example, snRNAseq of cortical gray matter and subcortical white matter of MS and control tissues found selective vulnerability of CUX2-positive excitatory neurons in upper cortical layers in MS (Schirmer et al., 2019; Table 1, PRJNA544731). Interestingly, sex-specific gene expression changes occur in prefrontal or entorhinal cortical neurons in AD (Belonwu et al., 2022; Table 1, GSE11882, GSE138852). Bulk RNA sequencing of laser-captured motor neurons from ALS and control lumbar spinal cords identified specific gene signatures enriched in immune cell functions (Krach et al., 2018; Table 1, GSE76220). Molecular screening of vulnerable vs. resistant motor neurons in SOD1 mice identified resistance and vulnerability profiles, including, e.g., matrix metalloproteinase-9 as marker and cause of degeneration of vulnerable fast motor neurons (Kaplan et al., 2014; Table 1, GSE52118). SnRNAseq of human postmortem midbrain tissues in idiopathic PD revealed the presence of a specific cluster of dysfunctional dopaminergic neurons characterized by CADPS2 overexpression and low thyroid hormone levels (Smajić et al., 2022; Table 1,GSE157783). Single cell transcriptomic atlas of the α-syn-A53T PD mouse model depicted dysregulation of ion channel components and glutamatergic signaling (Zhong et al., 2021; Table 1, CNP0000892). Cell type-specific transcriptomics of human HD and mouse models of HD depicted mitochondrial dysfunction accompanied by mitochondrial RNA release and activation of innate immune pathways in striatal spiny neurons (Lee et al., 2020; Table 1, GSE152058).

## Transcriptomics of microglia under physiology and disease

Microglia originate from precursors that migrate from the yolk sac to the developing CNS early during embryogenesis and undergo maturation postnatally thanks to the interaction with other cell types including astrocytes (Bennett et al., 2016; Kracht et al., 2020; Table 1, GSE141862, PRJNA307271). Transcriptional profiles for early microglia (until embryonic day 14), pre-microglia (from embryonic day 14 to a few weeks after birth), and adult microglia (from a few weeks after birth onward) were described for mouse cells (Matcovitch-Natan et al., 2016; Table 1, GSE79819). Bulk transcriptome profiling of rodent microglia throughout the lifespan and parallel comparison with peripheral macrophages demonstrated that phenotypic differentiation between microglia and peripheral macrophages is age-dependent and that peripheral macrophages may express some of the most commonly described microglia-specific markers early during development, such as Fcrls, P2ry12, Tmem119, and Trem2 (Grassivaro et al., 2020; Table 1, E-MTAB-8059). On the other hand, scRNAseq of mouse embryonic, juvenile and adult microglia from distinct CNS regions emphasized region-dependent microglia specification, with major changes between juvenile and adult microglia in the cortex and hippocampus but not cerebellum (Masuda et al., 2019; Table 1, GSE120629, GSE120747, GSE124335). Similarly, RNA sequencing of human white or gray matter microglia highlighted major regional differences, with NF-κB-related transcripts higher in white matter and type-I interferon transcripts higher in gray matter (van der Poel et al., 2019; Table 1, GSE111972). Sex differences were evident in microglia from the adult brain and conserved when cells were transplanted in brains of the opposite sex (Villa et al., 2018; Table 1, SRP104620). Molecular correlates for ageing are present in microglia and regard mostly inflammatory and immunomodulatory genes (Pan et al., 2020; Table 1, GSE137028).

Regional heterogeneity of microglia is described in MS lesions, where white matter microglia upregulate lipid metabolism gene expression while gray matter microglia display high levels of genes associated with glycolysis and iron homeostasis. On the contrary, expression of homeostatic genes, such as *P2RY12* and *TMEM119,* is unaltered in the normal appearing white matter close to the MS lesions, suggesting preservation of microglia homeostatic function at an early phase of lesion formation (van der Poel et al., 2019; Table 1, GSE111972). A specific cluster of disease associated microglia appears to be associated with human AD and its animal models (Wang, 2021). This cluster displays downregulation of microglia homeostatic genes (e.g., P2ry12, CX3CR1 and Tmem119) and major levels of known AD risk genes (e.g., ApoE, Tyrobp and Trem2; Keren-Shaul et al., 2017; Rothman et al., 2018; Wang et al., 2018; Marttinen et al., 2019; Hashemiaghdam and Mroczek, 2020; Table 1, GSE98969, GSE123467). Alterations in microglia Trem2-ApoE pathway is shared among animal models for MS, AD and ALS (Krasemann et al., 2017; Table 1, GSE101689). In idiopathic PD microglia revealed a pro-inflammatory profile when examined through snRNAseq of postmortem tissues (Smajić et al., 2022; Table 1, GSE157783). Similarly, inflammation-related genes (INFA, STAT1, STAT3) characterized microglia of mouse models of HD (Benraiss et al., 2021; Table 1, GSE154131).

## Useful atlases for CNS transcriptomics

In addition to the mentioned publicly available raw datasets, which require ex novo data processing and filtering, there are some useful free atlases, which allow users to retrieve already processed data at the single gene and cell level. Here are some examples: single cell transcriptome atlases of the developing mouse and human spinal cord,^1^ mouse spinal cord atlas described in Russ et al. (2021), human brain transcriptome databases as summarized in Keil et al. (2018), transcriptional landscape of the mammalian brain as at https://portal.brain-map.org/explore/transcriptome, and disease specific atlases of the CNS, e.g., the MS brain lesion atlas^2^ and the RADC Research Resource Sharing Hub for AD.^3^

## Conclusion and perspectives

This review represents an easy guide and a readily available reference of the available transcriptomics datasets for human and mouse CNS. In addition, it highlights the spatiotemporal and sexual heterogeneity of tissues and cell types across development, adulthood, aging, physiology, and neurodegeneration. It also serves as a gateway to identify appropriate *ex vivo* CNS studies in distinct neuroscience fields and empower future intra- and inter-disciplinary research endeavors so to highlight, e.g., how different diseases manifest in the same tissue or how the same disease affects distinct CNS areas, along ageing and/or according to sex.

It is important to note that most of the studies are limited to transcriptional descriptions, so that mechanisms and alterations in cellular functions and cell–cell crosstalk may be hypothesized but still require proper *in vitro* and *in vivo* validations. To this goal, rabies-activated brain-wide imaging and dissection with sequencing (RABID)-seq is an interesting, recently developed technique that combines rabies virus-based tracing, imaging, and scRNA-seq to associate transcriptional phenotypes with neural circuits and cellular interactions in the CNS.

Further, some of the transcriptional descriptions suggest interactions between cells of the nervous and immune systems. Also, immune surveillance of the CNS takes place under homeostatic conditions and pathological neuroinflammatory conditions, such as those occurring in multiple sclerosis, may lead to recruitment of immune cells from the circulation into CNS parenchyma (Mapunda et al., 2022). Thus, transcriptomics of CNS innate and adaptive immunity especially under pathological conditions may lead to the acquisition of additional information about cellular synergies important to design therapeutic strategies modulating immune responses and restoring CNS tissue homeostasis. This knowledge may also provide the basis for the development of biomarkers for specific CNS states and of therapeutic targets either specific to or shared among neurological conditions. Crossing information derived from distinct transcriptional studies will help the neuroscience community to make new discoveries at unprecedented speed and depth. A very good example of data integration is scREAD (Single-Cell RNA-Seq Database for Alzheimer’s Disease), which collected and analyzed scRNA-Seq and snRNA-Seq data sets relative to human postmortem brain tissues with AD and mouse models with AD pathology, thus providing control atlas generation, cell type prediction, identification of differentially expressed genes, and identification of cell-type specific regulons (Jiang et al., 2020). Moreover, the combination of distinct RNA-seq techniques may allow for overcoming the limits of each approach leading to more comprehensive descriptions of CNS state. This information represents the essential standard for validation of *in vitro* and *in vivo* models, which do have the complementary role of providing key mechanistic clues for brain function.

**BOX 1**

Multiple sclerosis (MS) and its animal models

*MS*, also known as encephalomyelitis disseminata, is a chronic CNS disorder with onset in young adulthood and female prevalence, presenting with multiple focal lesions characterized by inflammation, demyelination and neurodegeneration and leading to important neurological disability (McGinley et al., 2021).

*Experimental autoimmune encephalomyelitis (EAE)*: T cell- mediated autoimmune disease of the central nervous system with clinical and neuropathological similarities to MS. It is induced by active immunization with myelin extracts, purified myelin proteins, or immunogenic myelin peptides, or by adoptive transfer of myelin-reactive T lymphocytes (Lassmann and Bradl, 2017).

*Cuprizone model*: a model for non-immune mediated CNS demyelination. Young mice are fed with the copper chelator cuprizone, leading to oligodendrocyte death and subsequent demyelination. Spontaneous remyelination may occur after withdrawal of cuprizone diet (Lassmann and Bradl, 2017).

Alzheimer’s disease (AD) and its animal model

*AD* is a neurodegenerative CNS disorder characterized by b-amyloid positive extracellular plaques and tau-positive intracellular neurofibrillary tangles. It affects mainly the aged female population, presents initially with amnestic cognitive impairment and later with dementia (Knopman et al., 2021).

*5XFAD* (Familiar Alzheimer Disease) *mice*: they overexpress five human AD-linked mutations, three in the amyloid precursor protein (APP) 695 gene [APP K670N/M671L, I716V, V717I], and two more mutations in the PSEN1 gene [M146L, L286V]. The expression of the 5xFAD transgenes is driven by the neuron specific Thy1 promoter. Similarly to AD, such mice accumulate b-amyloid in the CNS and experience memory impairment (Oblak et al., 2021).

Parkinson’s disease (PD) and its animal models

*PD* is a CNS degenerative disorder affecting the aged population, with male prevalence, starting with bradykinesia and tremor and leading to gait disorder and dementia. PD neuropathology is characterized by neuronal loss in the substantia nigra, which causes striatal dopamine deficiency, and by intracellular inclusions containing aggregates of a-synuclein (Poewe et al., 2017).

*α-syn mouse model*: human α-syn overexpression mouse model that recapitulates some of the pathological features of PD in terms of progressive aggregation of human α-syn, impaired striatal dopamine fiber density, and an age-dependent motor deficit consistent with an impaired dopamine release (Hansen et al., 2013).

*MPTP-induced mouse model*: 1-methyl-4-phenyl-1,2,3,6-tetrahydropyridine (MPTP) yields large variations in nigral cell loss, striatal dopamine loss and behavioral deficits. Motor deficits do not fully replicate those seen in PD (Meredith and Rademacher, 2011).

Amyotrophic lateral sclerosis (ALS) and Its animal model

*ALS* is a rare neurological disease with male prevalence characterized by the degeneration of both upper and lower motor neurons which leads to muscle weakness and eventual paralysis. Mutations in SOD1 gene are associated with familial ALS and lead to neuronal accumulation of misfolded SOD1 proteins (Hardiman et al., 2017).

*SOD1 G93A mice*: they express the human G93A mutant form of human SOD1 under the control of SOD1 promoter. This leads to neurotoxicity in several ways, thus causing paralysis in one or more limbs within a few weeks of age (Hardiman et al., 2017).

Huntington’s disease (HD) and its animal model

*HD* is a rare neurodegenerative disease caused by a dominantly inherited CAG trinucleotide repeat expansion in the huntingtin gene leading to protein aggregate formation followed by neuronal dysfunction and death starting in the striatum (McColgan and Tabrizi, 2018).

*R6/2 mice*: they express exon 1 of the human HD gene with around 150 CAG repeats under the human huntingtin promoter and reproduce huntingtin accumulation in neurons and progressive brain atrophy (Bondulich et al., 2021).

## Author contributions

CF: conceptualization. CF: supervision and funding acquisition. SA and CF: investigation and writing. All authors contributed to the article and approved the submitted version.

## Funding

The study was funded by Italian Ministry of Health (RF-2018-12367731 to CF), FISM (Fondazione Italiana Sclerosi Multipla, grant number 2016/R/14 to CF) and cofinanced with the 5 per mille public funding.

## Conflict of interest

The authors declare that the research was conducted in the absence of any commercial or financial relationships that could be construed as a potential conflict of interest.

## Publisher’s note

All claims expressed in this article are solely those of the authors and do not necessarily represent those of their affiliated organizations, or those of the publisher, the editors and the reviewers. Any product that may be evaluated in this article, or claim that may be made by its manufacturer, is not guaranteed or endorsed by the publisher.

## Footnotes

^1^https://data.humancellatlas.org/explore/projects/6621c827-b57a-4268-bc80-df4049140193

^2^http://msatlas.compbio.sdu.dk/

^3^https://www.radc.rush.edu/

## Abbreviations

AD: Alzheimer’s disease
ALS: amyotrophic lateral sclerosis
CNS: central nervous system
EAE: experimental autoimmune encephalomyelitis
HD: Huntington disease
MS: multiple sclerosis
OL: oligodendrocyte
OPC: oligodendrocytes precursor cell
PD: Parkinson’s disease
scRNA seq: single cell RNA sequencing
snRNA seq: single nucleus RNA sequencing
